# Cobalamin Deficiency in Elderly Patients: A Personal View

**DOI:** 10.1155/2008/848267

**Published:** 2008-05-26

**Authors:** Emmanuel Andrès, Thomas Vogel, Laure Federici, Jacques Zimmer, Ecaterina Ciobanu, Georges Kaltenbach

**Affiliations:** ^1^Department of Internal Medicine, Diabetes, and Metabolic Diseases, University Hospital of Strasbourg, 67091 Strasbourg, France; ^2^Department of Internal Medicine and Geriatrics, University Hospital of Strasbourg, 67091 Strasbourg, France; ^3^Laboratoire d'Immunogénétique-Allergologie, Centre de Recherche Public de la Santé (CRP-Santé) de Luxembourg, 1526 Luxembourg, Luxembourg

## Abstract

Cobalamin (vitamin B12) deficiency is particularly common in the elderly (>65 years of age) but is often unrecognized because its clinical manifestations are subtle; however, they are also potentially serious, particularly from a neuropsychiatric and hematological perspective. In the elderly, the main causes of cobalamin deficiency are pernicious anemia and food-cobalamin malabsorption. Food-cobalamin malabsorption syndrome is a disorder characterized by the inability to release cobalamin from food or its binding proteins. This syndrome is usually caused by atrophic gastritis, related or unrelated to *Helicobacter pylori* infection, and long-term ingestion of antacids and biguanides. Management of cobalamin deficiency with cobalamin injections is currently well documented but new routes of cobalamin administration (oral and nasal) are being studied, especially oral cobalamin therapy for food-cobalamin malabsorption.

## 1. Introduction

Cobalamin or vitamin B12 deficiency is common in elderly patients [1] but is often unrecognized or not investigated because the clinical manifestations of cobalamin deficiency are subtle. However, the complications of cobalamin deficiency, particularly the neuropsychiatric and hematological [1–4], are potentially serious and therefore require investigation in all patients who present with vitamin or nutritional deficiency. Classic disorders such as pernicious anemia are the cause of cobalamin deficiency in only a limited number of patients, especially elderly patients [4]. A more common problem is food-cobalamin malabsorption, a disorder characterized by the inability to release cobalamin from food or its binding proteins [4]. This review summarizes the current knowledge on cobalamin deficiency, with a particular focus on food-cobalamin malabsorption and oral cobalamin therapy.

## 2. Definition of Cobalamin Deficiency

**Figure 1:**
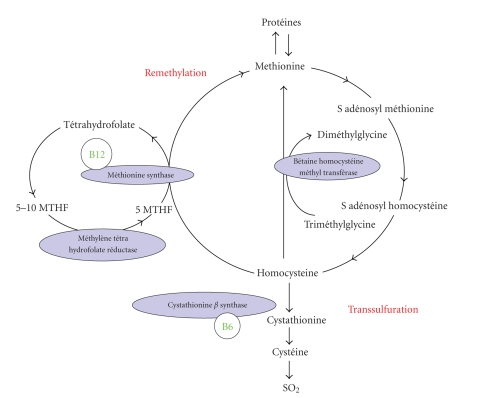
Cellular impact of cobalamin deficiency.

Literature of the last ten years has provided several definitions of cobalamin deficiency [5–7]. The definitions of cobalamin deficiency used in this review are shown in Table 1 [7, 8]. To date, cobalamin deficiency is often defined in terms of the serum concentration of cobalamin and of homocysteine and methyl malonic acid, two components of the cobalamin metabolic pathway, (Figure 1) but in clinical practice, no single test has emerged as the gold standard for diagnosis of cobalamin deficiency especially in elderly patients. Moreover, the major diagnostic challenge remains patients who develop subtle cobalamin deficiency, often without hematological abnormalities (usefulness of an early treatment to prevent irreversible neurological damages) [4]. In the future, new serum cobalamin assay kits (e.g., the holotranscobalamin assay kit) might perhaps replace older assay kits and should become the standard for testing [6, 9].

## 3. Epidemiology of Cobalamin Deficiency

Epidemiological studies show that in the general population of industrialized countries, cobalamin deficiency has a prevalence of around 2 to 20%, depending on the definition of cobalamin deficiency used [4, 9]. The Framingham study demonstrated a prevalence of 12% among elderly people living in the community [10]. Other studies focusing on elderly people, particularly those who are in institutions or who are sick and malnourished, have suggested a higher prevalence of at least 30% [11, 12]. Using the definition in Table 1 (serum cobalamin levels <150 pmol/L [<200 pg/mL] on 2 separate occasions), we found that cobalamin deficiency had a prevalence of 5% in a group of patients followed or hospitalized in a tertiary reference hospital [8]. We also documented that around 4% of the anemia were related to a cobalamin deficiency in a population of 300 consecutive anemia hospitalized in our department (tertiary reference center) [8]. In the NHANES III study, 34% of all anemia in elderly patients is caused by folate, cobalamin, or iron deficiency, alone or in combination (nutritient-deficiency anemia) [8].

## 4. Cobalamin Metabolism and Function

Cobalamin metabolism is complex and is made up of many processes, defects in any one of which can lead to cobalamin deficiency [4, 13–15]. The different stages of cobalamin metabolism and corresponding causes of cobalamin deficiency are shown in Table 2. Once metabolized, cobalamin is a cofactor and coenzyme for many biochemical reactions, including DNA synthesis, methionine synthesis from homocysteine, and conversion of propionyl into succinyl coenzyme A from methyl malonate [4, 9]. In a clinical setting, cobalamin absorption is measured imperfectly by the Schilling test [4, 8]. A typical Western diet contributes 3–30 *μ*g of cobalamin per day [13, 15] toward the recommended dietary allowance of 2.4 *μ*g/day for adults and 2.6 to 2.8 *μ*g/day during pregnancy [16]. The 5–10 year delay between the onset of cobalamin deficiency and the development of clinical illness is directly attributable to hepatic stores of cobalamin (>1.5 mg) and the enterohepatic cycle [4, 13]. Between 1–5% of free cobalamin (or crystalline cobalamin) is absorbed along the entire intestine by passive diffusion. This absorption explains the mechanism underlying oral treatment of cobalamin deficiencies [17, 18].

## 5. Classical Causes of Cobalamin Deficiency

In elderly patients, cobalamin deficiency is classically caused by pernicious anemia and food-cobalamin malabsorption [1, 11, 14]. The principal characteristics of pernicious anemia have been reported in detail in several reviews [19–21]. Diagnosis of pernicious anemia is based on the presence of (1) intrinsic factor antibodies in serum (specificity: >98%, sensibility: around 50%) and/or (2) autoimmune atrophic gastritis (presence of *Helicobacter pylori infection* in gastric biopsies is an exclusion factor) [15, 19]. Cobalamin deficiency caused by dietary deficiency or malabsorption is rare. Dietary causes of deficiency are limited to elderly people who are already malnourished. This mainly concerns elderly patients living in institutions or in psychiatric hospitals [4, 13]. Since the 1980s, the malabsorption of cobalamin has become rarer, owing mainly to the decreasing frequency of gastrectomy and surgical resection of the terminal small intestine [4, 14]. Several disorders commonly seen in gastroenterology practice might, however, be associated with cobalamin malabsorption. These include deficiency in the exocrine function of the pancreas after chronic pancreatitis (usually alcoholic), lymphomas or tuberculosis (of the intestine), Crohn's disease, Whipple's disease, and uncommonly celiac disease [11, 15].

## 6. Food-Cobalamin Malabsorption

First, well-described by Carmel in 1995 [22], the food-cobalamin malabsorption is a syndrome characterized by the inability to release cobalamin from food or intestinal transport proteins, particularly in the presence of hypochlorhydria, in which the absorption of “unbound” cobalamin is normal. As various studies have shown [14, 22, 23], this syndrome is defined by cobalamin deficiency in the presence of sufficient food-cobalamin intake and normal Schilling test results, which rules out malabsorption or pernicious anemia. The principal characteristics of this syndrome are listed in Table 3. In theory, indisputable evidence of food-cobalamin malabsorption comes from using a modified Schilling test, which uses radioactive cobalamin bound to animal proteins (e.g., salmon, trout) and reveals malabsorption when the results of a standard Schilling test are normal [4, 14, 23].

Food-cobalamin malabsorption has been found to be the leading cause of cobalamin malabsorption, especially in elderly patients [4, 11, 22]. In our experience (300 patients with a documented cobalamin deficiency), food-cobalamin malabsorption accounts for about 60–70% of the cases of cobalamin deficiency in elderly patients, whereas pernicious anemia accounted for only 15–25% [14, 23]. Some authors have speculated about the reality and significance of cobalamin deficiency related to food-cobalamin malabsorption [4], because many patients have only mild clinical or hematological features. Several of our patients, however, [14] had significant features classically associated with pernicious anemia, including polyneuropathy, confusion, dementia, medullar-combined sclerosis, anemia, and a pancytopenia. Nevertheless, the partial nature of this form of malabsorption might produce a more slowly progressive depletion of cobalamin than does the more complete malabsorption engendered by disruption of intrinsic factor-mediated absorption. The slower progression of depletion probably explains why mild preclinical deficiency is associated with food-cobalamin malabsorption more often than with pernicious anemia [4, 14].

Food-cobalamin malabsorption is caused primarily by atrophic gastritis [14]. Achlorhydria hampers the extraction of cobalamin from protein food sources. Over 40% of patients older than 80 years of age have gastric atrophy that might or might not be related to *Helicobacter pylori* infection [11, 24]. Other factors that contribute to food-cobalamin malabsorption in elderly people include chronic carriage of *H. pylori* and intestinal microbial proliferation (in which case cobalamin deficiency can be corrected by antibiotic treatment) [24, 25]; long-term ingestion of antiacids, including H2-receptor antagonists and proton-pump inhibitors [26, 27], particularly among patients with Zollinger-Ellison syndrome [28, 29], and biguanides (metformin) [30–32]; chronic alcoholism; surgery or gastric reconstruction (e.g., bypass surgery for obesity); partial pancreatic exocrine failure [4, 14], and Sjögren's syndrome or systemic sclerosis [33] (Table 3). In a series of 92 elderly patients (mean age: 76 years) with food-cobalamin malabsorption [14], we have reported at least one of these associated conditions or agents in 60% of the patients. These conditions mainly include atrophic gastritis (±*H. pylori* infection) in 30% of the patients and long-term metformin or antacid intake in 20% of the elderly patients.

## 7. Clinical Manifestations of Cobalamin Deficiency

The primary clinical manifestations of cobalamin deficiency are described in Table 4. They are highly polymorphic and of varying severity ranging from common sensory neuropathy and isolated anomalies of macrocytosis and hypersegmentation of neutrophils, to severe disorders, including combined sclerosis of the spinal cord, hemolytic anemia, and even pancytopenia [2, 14, 34–36]. In the aforementioned series of 92 patients with food-cobalamin malabsorption [14], we have found at least one clinical feature or hematological abnormalities in, respectively, 70% and 76% of the patients. Cobalamin deficiency appears to be more common among patients who have a variety of chronic neurologic conditions such as dementia, Alzheimer's disease, stroke, Parkinson's disease, and depression, although it is unclear if these are causal relationships [4, 37]. In our own studies in which we administered cobalamin to patients with dementia, improvement was not observed [8, 14]. Other studies have had similar results [1, 2, 9]. At this time, a causal role of cobalamin in these conditions remains speculative.

## 8. Classical Treatment of Cobalamin Deficiency

The classic treatment for cobalamin deficiency, particularly when the cause is not dietary deficiency, is parenteral administration—in most countries intramuscular injection—of this vitamin (in the form of cyanocobalamin and, more rarely, hydroxy or methyl cobalamin) [1, 17, 18, 34]. However, traditions concerning both dose and schedule of administration vary considerably. In France, the recommended practice is to build up the tissue stores of the vitamin quickly and correct serum cobalamin hypovitaminosis, particularly in the case of pernicious anemia. The treatment involves the administration of 1000 *μ*g of cyanocobalamin per day for 1 week, followed by 1000 *μ*g per week for 1 month, followed by 1000 *μ*g per month, normally for the rest of the patient's life [11, 19]. In USA and UK, dosages ranging from 100 to 1000 *μ*g per month [or every 2-3 months when hydroxocobalamin is given] are used during the rest of the patient's life [4, 17]. Hydroxocobalamin may have several advantages due to a better tissular retention and storage. Additionally, recent works concern oral cobalamin therapy through food fortification [3, 11].

## 9. Oral Cobalamin Therapy

Since cobalamin is absorbed by intrinsic factor-independent passive diffusion (1% of oral cobalamin), daily high-dose oral cyanocobalamin can induce and maintain remissions in patients with megaloblastic anemia [15]. In cases of cobalamin deficiency other than those caused by nutritional deficiency, alternative routes of cobalamin administration have been used: oral [17, 18, 38–44] and nasal [45, 46]. These other routes of administration have been proposed as a way of avoiding the discomfort, inconvenience, and cost of monthly injections. Our working group has developed an effective oral treatment of food-cobalamin malabsorption [40–43] and for pernicious anemia [47] using crystalline cobalamin (cyanocobalamin). Our principal studies of oral cobalamin treatment (open, not randomized studies) are described in Table 5 [40–43, 47]. These data confirm the previously reported efficacy of oral crystalline cyanocobalamin, especially in food-cobalamin therapy [18, 36, 38]. All of our patients who were treated orally corrected their cobalamin levels and at least two-thirds corrected their hematological abnormalities [40–43, 47]. Moreover, one-third of patients experienced a clinical improvement on oral treatment. In most cases of food-cobalamin malabsorption, “low” cobalamin doses (i.e., 125–1000 *μ*g of oral crystalline cyanocobalamin per day) were used. These data is in accordance with the results of the two prospective randomized-controlled studies comparing oral cobalamin with intramuscular cobalamin therapy [17, 39]. A systematic review of randomized-controlled trials by the *Vitamin B12 Cochrane Group* supports the efficacy of oral cobalamin therapy, with a dose between 1000 and 2000 *μ*g given initially daily and then weekly [48]. In this analysis, serum cobalamin levels increased significantly in patients receiving oral cobalamin and both groups of patients (receiving oral and intramuscular treatment) had neurological improvement. The Cochrane group concludes that daily oral therapy “may be as effective as intramuscular administration in obtaining short term haematological and neurological responses in cobalamin deficient patients” [48]. Nevertheless to our knowledge, the effect of oral cobalamin treatment in patients presenting severe neurological manifestations has not yet been adequately documented. Thus until this has been done parenteral cobalamin therapy is still to be recommended for such patients. In a randomized, parallel-group, double-blind, dose-finding trial, Eussen et al. showed that the lowest dose of oral cyanocobalamin required to normalize mild cobalamin deficiency is more than 200 times the recommended dietary allowance of approximately 3 *μ*g daily (i.e., >500 *μ*g per day) [49]. The procedure for oral cobalamin treatment has, however, not been completely validated yet in real life, particularly the long-term efficacy [50]. To date, as several authors suggest, oral cobalamin therapy remains one of “medicine's best kept secrets” [51]. Since loading doses of cobalamin far exceed physiologic requirements, clinical responses may result from pharmacologic effects on either cobalamin-related processes or on cellular functions completely unrelated to the known biochemical actions of cobalamin [52]. As a result, blood cobalamin, methylmalonic acid and homocysteine values often fail to predict whether or not a patient will respond to cobalamin therapy [53]. Nevertheless, the following can be proposed: ongoing supplementation until associated disorders are corrected (e.g., by halting the ingestion of the offending medication or exogenosis, or by treating *H. pylori* infection or pancreatic exocrine failure), lifelong administration or, when applicable, sequential administration [54].

## Figures and Tables

**Table 1:** Definitions of cobalamin (vitamin B12) deficiency [5–7].

(i) Serum cobalamin levels <150 pmol/L and clinical features and/or hematological anomalies related to cobalamin deficiency
(ii) Serum cobalamin levels <150 pmol/L (<200 pg/mL) on 2 separate occasions
(iii) Serum cobalamin levels <150 pmol/L and total serum homocysteine levels >13 *μ*mol/L or methylmalonic acid levels >0.4 *μ*mol/L (in the absence of renal failure and folate and vitamin B6 deficiencies)
(iv) Low serum holotranscobalamin levels <35 pmol/L

**Table 2:** Stages of cobalamin metabolism and corresponding causes of cobalamin deficiency [13, 15].

Stages and factors involved in cobalamin metabolism	Causes of cobalamin deficiency
Ingestion of food	Strict vegetarianism (patients who are sick in institutions or in psychiatric hospitals)
Digestion, which involves haptocorrin, gastric secretions (HCl and pepsin), intrinsic factor, pancreatic and biliary secretions, and the enterohepatic cycle	Gastrectomy, pernicious anemia, and food-cobalamin malabsorption
Absorption, which brings into play intrinsic factor and cubilin	Ileal resection, malabsorption, pernicious anemia, and food-cobalamin malabsorption
Transportation by transcobalamins	Congenital deficiency in transcobalamin II
Intracellular metabolism by various intracellular enzymes	Congenital deficiency in various intracellular enzymes

HCl = hydrochloric acid.

**Table 3:** Food-cobalamin malabsorption syndrome [4, 14, 15].

Criteria for food-cobalamin malabsorption	Associated conditions or agents
– Low-serum cobalamin (vitamin B12) levels	– Gastric disease: atrophic gastritis, type A atrophic gastritis, gastric disease associated with *Helicobacter pylori* infection, partial gastrectomy, gastric by-pass, and vagotomy
– Normal results of Schilling test using free cyanocobalamin labeled with cobalt-58, or abnormal results of derived Schilling test^‡^	– Pancreatic insufficiency: alcohol
– No anti-intrinsic factor antibodies	– Gastric or intestinal bacterial overgrowth: achlorhydria, tropical sprue, Ogylvie's syndrome, and HIV
– No dietary cobalamin deficiency	– Drugs: antacids (H2-receptor antagonists and proton-pump inhibitors) or biguanides (metformin)
	– Alcohol abuse
	– Sjögren's syndrome, systemic sclerosis
	– Haptocorrine deficiency
	– Ageing or idiopathic

^‡^Derived Schilling tests use food-bound cobalamin (e.g., egg yolk, chicken, and fish proteins).

**Table 4:** Main clinical features of cobalamin deficiency [2, 4, 14, 15, 34–36].

Hematological manifestations	Neuro-psychiatric manifestations	Digestive manifestations	Other manifestations
– Frequent: macrocytosis, hypersegmentation of the neutrophils, aregenerative macrocytary anemia, LDH and bilirubin elevation, medullary megaloblastosis “(blue spinal cord)”	– Frequent: polyneurites (especially sensitive ones), ataxia, Babinski's phenomenon	– Classic: Hunter's glossitis, jaundice, LDH and bilirubin elevation “(intramedullary destruction)”	– Under study: atrophy of the vaginal mucosa and chronic vaginal and urinary infections (especially mycosis), hypofertility and repeated miscarriages (connection with cobalamin deficiency under study), venous thromboembolic disease, angina (hyperhomocysteinemia), osteoporosis
– Rare: isolated thrombocytopenia and neutropenia, pancytopenia	– Classic: combined sclerosis of the spinal cord	– Debatable: abdominal pain, dyspepsia, nausea, vomiting, diarrhea, disturbances in intestinal functioning	
– Very rare: hemolytic anemia, thrombotic microangiopathy (presence of schistocytes)	– Rare: cerebellar syndromes affecting the cranial nerves including optic neuritis, optic atrophy, urinary, and/or fecal incontinence	– Rare: resistant and recurring mucocutaneous ulcers cobalamin deficiency	
	– Under study: changes in the higher functions, even dementia, stroke and atherosclerosis (hyperhomocysteinemia), parkinsonian syndromes, depression, multiple sclerosis		

**Table 5:** Experience of oral cobalamin therapy for food-cobalamin malabsorption in the university hospital of Strasbourg, France.

Study characteristics (number of patients)	Therapeutic modalities	Results	
Open prospective study of well-documented cobalamin deficiency related to food-cobalamin malabsorption (*n* = 10)	Oral crystalline cyanocobalamin: 650 *μ*g per day during at least 3 months	– Normalization of serum cobalamin levels in 80% of the patients	[41]
		– Significant increase of hemoglobin (Hb) levels (mean of 1.9 g/dL) and decrease of mean erythrocyte cell volume (ECV) (mean of 7.8 fL)	
		– Improvement of clinical abnormalities in 20% of the patients	

Open prospective study of low-cobalamin levels not related to pernicious anemia (*n* = 20)	Oral crystalline cyanocobalamin: between 1000 *μ*g per day during at least 1 week	– Normalization of serum cobalamin levels in 85% of the patients	[42]
		– No adverse effect	

Open prospective study of well-documented cobalamin deficiency related to food-cobalamin malabsorption (*n* = 30)	Oral crystalline cyanocobalamin: between 1000 and 250 *μ*g per day during 1 month	– Normalization of serum cobalamin levels in 87% of the patients	[40]
		– Dose effect: effectiveness dose of cobalamin ≥500 *μ*g per day	
		– No adverse effect	

Open prospective study of low-cobalamin levels not related to pernicious anemia (*n* = 30)	Oral crystalline cyanocobalamin: between 1000 and 125 *μ*g per day during at least 1 week	– Significant increase of Hb levels (mean of 0.6 g/dL) and decrease of ECV (mean of 3 fL); normalization of Hb levels and ECV in 54% and 100% of the patients, respectively	[43]
		– Normalization of serum cobalamin levels in all patients with at least a dose of vitamin ≥250 *μ*g per day	
		– Dose effect: effectiveness dose of cobalamin ≥500 *μ*g per day	
		– No adverse effect	

Open prospective study of low cobalamin levels related to pernicious anemia (*n* = 10)	Oral crystalline cyanocobalamin: 1000 *μ*g per day during at least 3 months	– Significant increase of serum cobalamin levels in 90% of the patients (mean of 117.4 pg/mL)	[47]
		– Significant increase of Hb levels (mean of 2.45 g/dL) and decrease of ECV (mean of 10.4 fL)	
		– Improvement of clinical abnormalities in 30% of the patients	
